# The impact of FinTech firms on bank financial stability

**DOI:** 10.1007/s10660-022-09595-z

**Published:** 2022-08-03

**Authors:** Md Safiullah, Sudharshan Reddy Paramati

**Affiliations:** 1 RMIT University, School of Economics, Finance and Marketing, Melbourne, Australia; 2 School of Business, University of Dundee, Dundee, UK

**Keywords:** G21, G23, G24, FinTech firms, Bank financial stability, Emerging market

## Abstract

This study is the first to examine the impact of FinTech firms on bank financial stability. Using a sample of 26 banks from an emerging market (Malaysia), over the period 2003–2018, we find that the development of FinTech firms over time increases bank financial stability. We uncover further evidence that FinTech firms’ impact on bank financial stability holds when we conduct sub-sample analyses by bank size, bank type (Islamic vis-à-vis conventional), and level of corporate governance. The results are robust to alternative model specifications, measures of financial stability, and FinTech.

## Introduction

Financial technology (FinTech) companies have become increasingly important sources of financial services in both developed and emerging markets [8, 18], Ernst & [15]. Global investment in FinTech increased from $9.28b in 2008 to $168b in 2018.^1^ Interestingly, of the world’s top 100 leading FinTech cities, almost half are located in emerging markets. The expansion of this sector is primarily a technological response to the deficiencies of traditional banks and other financial service providers, which experienced financial instability in the aftermath of the 2007–2008 global financial crisis (GFC). Furthermore, the COVID-19 pandemic and the resultant demand for contactless banking transactions created opportunities for this sector to grow more rapidly [12]. FinTech firms offer a broad array of disrupted and innovative financial services [46]. Fintech service providers use technology to disrupt financial services historically offered by existing banks and simultaneously invent new financial services (e.g., peer-to-peer [P2P] lending and mobile phone payments. By doing so, they compete with banks in similar market segments and businesses but engage with a wider customer base, and offer easily accessible and low-cost financial services [5, 17, 18, 30]. These benefits to customers make FinTech firms a strong competitive force in the banking industry. The effect of market rivalry on bank stability has been widely studied [3, 4, 11, 19]. However, whether—and to what extent—these new entrants impact bank financial stability is unexplored. Therefore, this study aimed to address this research question.

The motivation for conducting this study was based on three important sources. First, no systematic empirical study has been conducted on this topic. This study fills this research gap. Second, we believe that this topic is important and worth empirically examining because the FinTech market is growing rapidly, and the banking industry is under market pressure to adopt sophisticated financial technologies in their transactions and services. This pressure has received significant attention in the banking industry, and consequently, FinTech-based financial services are being prioritized by banks [8, 36, 46]. However, introducing these new banking services merely to compete with new entrants at the cost of financial stability may jeopardize the entire banking industry. The financial services industry experienced a significant crisis in 2007–2009, and such financial instability may reoccur if there is unhealthy competition and if less objective-oriented FinTech services are added to the product base of banking services. Finally, despite Malaysia's significant growth in FinTech and dual-banking systems, there has been scant prior research on FinTech and banking. Our study adds to the literature and knowledge that FinTech firm development matters for bank financial stability.

The following question arises: How do FinTech firms affect bank financial stability? Besanko and Thakor [3] and Boyd and De Nicolo [4] theoretically argued that increased competition may decrease or improve banks’ financial stability. Building on their theoretical perspectives, we hypothesize that the increased competition caused by the rapid expansion of FinTech firms may lower market share and rents for banks from relationship lending, which may induce banks to make risky investments, thereby reducing financial stability. Nonetheless, FinTech firms may impose indirect pressure on banks to either adopt FinTech as part of their own services or engage FinTech service providers in their services, which may help banks operate efficiently, maintain profitability, and thereby remain financially stable. Thus, the effect of FinTech firms on bank financial stability is a *priori* indeterminate. In this study, we empirically determine the nexus between FinTech firms and bank stability.

Our contributions to the literature are threefold: First, this study is the first to examine FinTech firms’ impact on bank financial stability. Second, the present study is also the first to investigate this impact in the context of both Islamic and conventional banks. The former bank type follows Islamic law (Shariah) as a mode of operation, which prohibits dealing with interest (*riba*), uncertainty (*gharar)*, gambling (*mysir*), and other business activities that are deemed detrimental to people’s well-being [14, 42]. These differences between the two bank types motivate us to examine whether the impact of FinTech firms’ growth on bank stability differs between bank types. Third, we examine whether FinTech firms’ impact on bank stability differs based on the level of corporate governance. Arguably, FinTech firms are less governed and regulated and, therefore, undertake risky lending and investment activities [45]. This concern drives our interest in exploring whether the competition arising from the FinTech market equally affects bank stability—irrespective of the level of bank corporate governance. These three research issues have not been addressed in previous studies. Taken together, this research expands our knowledge base on FinTech firms’ impact—and their differential impact between bank types —on bank financial stability.

For the empirical investigation, we use pooled ordinary least squares (POLS) regression as the baseline research method. We examine both the contemporaneous and lagged effects of FinTech on banks’ financial stability. The use of lagged values helps reduce the concern of reverse causality and permits our variable of interest some time lag to affect bank financial stability. We further use the dynamic panel generalized method of moments (GMM) estimator, which allows us to account for unobserved heterogeneity, simultaneity, and dynamic endogeneity in our estimation. We use a sample of both Islamic and conventional banks from Malaysia for the 2003–2018 period. Our results show that the development of FinTech firms over time increases bank financial stability. We find robust evidence when we conduct subsample analyses by bank size, bank type (Islamic vs. conventional), and level of corporate governance. Our results are consistent with alternative model specifications, measures of financial stability, and FinTech.

This study has important implications for regulators, policymakers, and bank managers. It provides important insights for regulators to adopt strategies to promote FinTech development to ensure the banking industry’s financial stability. Our research can guide policy makers to promulgate FinTech related policies and ensure good governance, as FinTech development promotes banking stability. This study’s findings may also guide bank managers in both Islamic and conventional banks to augment FinTech-based financial services.

The remainder of this paper is organized as follows: Sect. 2 presents a literature review and hypothesis development. Section 3 reports on the data and methodology used. Section 4 presents the empirical results and a discussion. Section 5 presents the results of the robustness test. Finally, Sect. 6 concludes the study with policy implications and directions for future research.

## Literature review and hypothesis development

### Literature review

FinTech firms have received increasing attention in recent years owing to their rapid development and expansion across economies. The rise of FinTech firms has been welcomed by numerous observers who believe that new technological innovations in the financial industry have great potential to transform financial services by offering less expensive transactions, thus making these services more convenient and secure (e.g., [7, 8, 20]. Similarly, Philippon [39] argued that FinTech firms in the financial sector offer digital innovations and technology-enabled business model inventions, which significantly contribute in improving financial services to the wider communities.

An important model of FinTech innovations in the recent period is P2P lending, which has attracted great attention owing to its rapid expansion in emerging markets, such as China. The P2P lending transaction volume in China is the highest in the world, reaching nearly $550 billion in 2017. Importantly, the market diffusion rate is also ranked highest in China. Further, Huang [23] argued that the main drivers of P2P lending in China are a large supply of funds, greater market dispersion rate, and increasing demand for financial products.

Most importantly, P2P lending replaces the traditional banking system through an electronic marketplace that enables the brokerage of consumer loans between lenders and borrowers [24, 31]. However, notably, P2P lending in China is continuously evolving in a relatively underdeveloped legal and regulatory environment. Huang [23] and Milne and Parboteeah [32]^2^ documented that the P2P lending platform does not merely work as an intermediary to pull funds from retail investors and lend money to individual borrowers and small and medium-sized enterprises (SMEs); instead, it offers other value-added services, including checking the solvency of borrowers and loan ratings, managing payments, and providing investment advice to clients. Similarly, Tang [45] reported that U.S. P2P lending is a substitute for bank lending but complements small loans. Likewise, Fuster et al. [18] reported that FinTech firms in the U.S. offer more efficient mortgage lending services than other lenders—irrespective of clients’ level of financial access.

Despite increasing attention among scholars and practitioners of FinTech firms, the empirical literature on this research topic is scant. A recent study by Chen et al. [8] investigated the value of FinTech innovations using patent-filling data from 2003–2017. The authors used machine learning to identify and classify innovations in their underlying technologies. Their analysis showed that most FinTech innovations offer substantial value to innovators. Specifically, the authors stated that the Internet of Things, robo-advising, and blockchain are the most valuable innovations for the overall financial sector. Further, the authors highlighted that financial industries can avoid the negative impact of innovations by investing heavily in their own innovations.

Lee et al. [28] investigated whether the development of the FinTech industry influenced cost efficiency and technological adoption in the Chinese banking industry during the 2003–2017 period. Their evidence confirmed that Chinese state-owned banks not only operate with a less efficient technology but also have the lowest cost efficiency. However, the authors reported that FinTech development not only enhances the use of technology by the banks but also improves their cost efficiency. This dual benefit is more clearly observed in instances of market-supported service innovations.

In another study, Li et al. [30] examined the risk spillover between FinTech companies and conventional financial institutions during a period of rapid technological advancement. Using the U.S. financial and FinTech firms’ stock returns and the Granger causality framework, the authors investigated pairwise risk spillovers across quantiles. The main findings from the study indicated that FinTech firms’ risk spillover to financial institutions positively correlated with an increase in the systematic risk of financial institutions. Additionally, using a sample of 41 banks and FinTech firms in Indonesia, Phan et al. [36] examined whether the growth of FinTech firms negatively influences banking performance. Their main results demonstrated that FinTech firms’ growth negatively influences banks’ performance.

However, in a recent study, Sheng [44] explored the impact of FinTech firms on bank lending to SMEs in China. Using provincial Chinese banks’ lending data for the period from 2011 to 2018, the author confirmed that FinTech firms have significantly contributed in facilitating banking sector credit to SMEs. Noteworthily, FinTech firms’ influence on banking lending to SMEs is much stronger for large banks than for small banks. Evidently, although the literature on FinTech has been growing recently, it is still sparse.^3^

### Hypothesis development

**Fig. 1:**
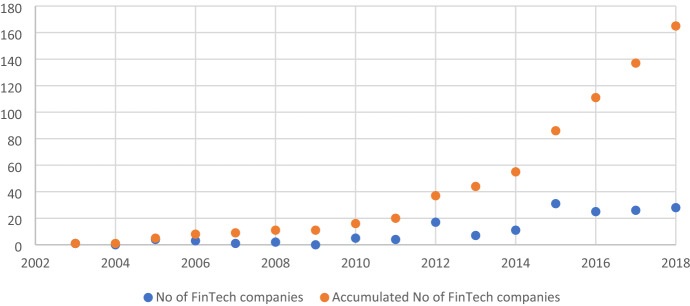
Number of FinTech companies over time (2003–2018)

The literature clearly shows a strong relationship between the growth of FinTech firms and the banking sector’s performance. For instance, Li et al. [29] reported a positive effect of FinTech firm growth on the U.S. banks’ share prices. From the emerging markets’ perspective, Phan et al. [36] demonstrated that FinTech firms’ growth reduced Indonesian banks’ performance, whereas Lee et al. [28], Sheng [44], Chen et al. [9], and Hu et al. [22] reported that FinTech firms’ development in China increases bank efficiency and reduces risk-taking, internal cash flow, and credit supply to SMEs. The findings of this strand of literature are mixed, which can be attributed to the differences in study contexts and topics. The other issue of whether FinTech firms increase or reduce bank financial stability remains unexplored. For example, some scholars (e.g., [3, 4] have theoretically argued that increasing competition may increase or decrease banks’ financial stability. Given this argument, we posit that increasing competition in the financial sector—due to FinTech firms’ rapid growth—may lower the banks’ profits from lending, thereby adversely affecting their share prices. Consequently, banks are forced to make risky investments, which will eventually lead to a reduction in their financial stability. Alternatively, due to the increasing presence of FinTech firms in the financial system, banks may be forced to adopt FinTech services in their banking business, which may eventually help them operate efficiently and maintain their customer base and revenues, thereby maintaining their financial stability. Given this backdrop, determining FinTech’s impact on bank financial stability is not possible without empirical investigation. Therefore, we empirically determine the relationship between FinTech firms and bank stability in the context of an emerging market—Malaysia, which is an ideal research context for this study for the following reasons: Malaysia is one of the fastest growing FinTech markets^4^ (see Fig. 1). Further, it is a prominent country with a dual-banking system that comprises Islamic as well as conventional banks [47]. Given the above arguments, we propose the following hypothesis:

#### H_1_

FinTech firms’ development improves banks’ financial stability.

## Data and methodology

### Sample and data

Our sample comprises 26 Islamic and conventional banks and 301 bank-year observations. We use 2003–2018 as our sample period because prior to 2003, FinTech firms in Malaysia were extremely limited (see Fig. 1). We use the Bankscope and Fitch Connect databases for bank-level financial data. We manually collect data on FinTech firms from FinTech Malaysia.^5^ Further, we collect bank-level board governance data from the annual reports of the respective banks. We use the World Development Indicator database for industry- and country-level variables.

### Measures of variables

Our primary measure of financial stability is the Z-score, with a higher Z-score indicating higher financial stability [27]. The Z-score is defined as follows: ${\text{Z - score}}_{{i,t}} = \left[ {{\text{ROA}}_{{i,t}} + {\text{CAR}}_{{i,t}} /{\text{SDROA}}_{{i,t}} } \right]$, where ROA is the return on assets, CAR is the capital-to-asset ratio, and SDROA is the standard deviation of ROA for bank $i$ at time$t$. The Z-score shows the number of standard deviations that a bank’s ROA would have to fall below its expected value to deplete equity and render the bank insolvent. Thus, a higher Z-score indicates greater financial stability. Following Fang et al. [16] and Safiullah [43], we further construct a relative measure of financial stability (hereafter, the RZ-score) using the stochastic frontier analysis technique. We estimate relative financial stability or the RZ-score using the stochastic frontier model, as in Fang et al. [16]. The stochastic stability frontier model and translog specification for the stochastic stability frontier model are specified as follows:

$$In(Z{\text{ }} - {\text{ }}score_{{it}} ) = Inf_{t} \left( {X_{{it}} } \right) + v_{{it}} + u_{{it}}$$

$$\left( {\frac{{Z{\text{ }} - {\text{ }}score_{{it}} }}{{w_{3} }}} \right) = \alpha _{0} + \sum\limits_{{g = 1}}^{3} {\alpha _{g} } \ln (y_{{git}} ) + \sum\limits_{{m = 1}}^{2} {\beta _{m} } \ln \left( {\frac{{w_{{mit}} }}{{w_{3} }}} \right) + \frac{1}{2}\sum\limits_{{g = 1}}^{3} {\sum\limits_{{h = 1}}^{3} {\gamma _{{gh}} } } \ln \left( {y_{{git}} } \right)\ln \left( {y_{{hit}} } \right) + \frac{1}{2}\sum\limits_{{m = 1}}^{2} {\sum\limits_{{n = 1}}^{2} {\delta _{{mn}} } } \ln \left( {\frac{{w_{{mit}} }}{{w_{3} }}} \right)\ln \left( {\frac{{w_{{nit}} }}{{w_{3} }}} \right) + {\text{ }}\sum\limits_{{g = 1}}^{3} {\sum\limits_{{m = 1}}^{2} {\theta _{{gm}} } } \ln (y_{{git}} )\ln \left( {\frac{{w_{{mit}} }}{{w_{3} }}} \right) + v_{{jit}} + u_{{jit}}$$

where $\ln Z - score_{it}$ is the logarithm of the Z-score for bank *i* in year *t*, ${y}_{g}$ refers to $g$ th output, and ${w}_{m}$ is $m$ th input price. To estimate the stability frontier model in Eq. (1) with this translog form, standard symmetric restrictions are applied to the second–order parameters or translog portion of the model as ${\gamma }_{gh}={\gamma }_{hg}$ and ${\delta }_{mn}={\delta }_{nm}$. Furthermore, to ensure price homogeneity, following Fang et al. [16], the Z-score, price of deposits $({w}_{1})$, and price of physical capital $({w}_{2})$ are normalized by the price of labor (${w}_{3}$). $\alpha$,$\beta$,$\gamma$,$\delta$, and $\theta$ are parameters to be estimated; ${v}_{it}$ contains the two–sided error $v\sim$ N (0,${\sigma }_{v}^{j2})$, capturing the effects of random error or statistical noise, which are independent of ${u}_{it}$. Finally, ${u}_{it}$ is a non–negative random variable, representing stability inefficiency. Relative stability is estimated by the following conditional expectation: ${SE}_{it}=E({e}^{{-u}_{it}}|{\varepsilon }_{it}$). We estimate the translog stability function using the maximum likelihood estimator (MLE). The MLE results are obtained with iterative procedures that comprise an estimation of OLS regression and a two-phase grid search for gamma,$\gamma$ (for details, see Coelli [10]). We define output variables and input prices as follows: total loans—total amount of customer loans; other earning assets—other earning assets comprising loans and advances to banks, other securities, derivatives (if any), and other investments; non-interest income—non-interest income comprising net gains (losses) on trading and derivatives, net gains (losses) on other securities, net insurance income, net fees and commissions, and other operating income; the price of deposits—the ratio of interest expenses to total deposits; the price of physical capital—the ratio of non-interest expenses (operating expenses minus personnel expenses) to total fixed assets; and the price of labor—the ratio of personnel expenses to total assets.

As suggested by Thakor [46], we use the following two proxies for FinTech firms, which are our variables of interest: the total number of all FinTech firms and number of FinTech firms closely related to banking services. Our second measure of FinTech addresses the possible concern that FinTech firms that are closely related to banking services may have a differential impact on bank financial stability than other FinTech firms.

Finally, we use control variables following the banking literature on the determinants of financial stability. Specifically, we use bank size measured as the logarithm of total assets. Further, bank age represents the number of years from the year of establishment to the end of 2018; the non-performing loans (NPL) ratio is the ratio of NPL to gross loans; equity capital ratio is the ratio of equity capital to total assets; assets growth is the annual growth rate of total assets; income diversity is the ratio of non-interest income to total operating income; ROA is the return on assets; bank concentration ratio is the assets of the three largest banks as a percentage of total banking industry assets; GDP growth rate is the annual growth rate of per capita GDP in percentage and bank-level corporate governance score; the governance score is the average of eight bank-level board of directors’ attributes—namely, board size, board independence, CEO duality, board busyness, board members’ financial expertise, audit committee size, audit committee chairman independence, and risk management committee size. The board governance score is constructed following the governance literature (e.g., [1]).

## Research method

To empirically test the assertion that FinTech firms impact bank stability, we employ the following regression model:

1 $${\text{FS}}_{it} = \alpha + \beta {\text{FinTech}}_{it} + \gamma X_{it} + \delta M_{t} + \varepsilon_{it }$$

where *i* and *t* refer to the bank and year, respectively. $FS$ stands for financial stability measures (Z- and RZ-scores). $FinTech$ refers to three proxies for FinTech firms (i.e., the number of all FinTech firms that are closely related and not related to the banking industry). $X$ indicates bank-level financial control variables, and $M$ represents industry- and macro-level control variables (as defined in Sect. 4.2). This model was used as the baseline. We further include GFC in our baseline model to investigate whether FinTech firms’ impact on bank financial stability holds, controlling for the GFC. Our extended model is as follows:

2 $$FS_{it} = \alpha + \beta {\text{FinTech}}_{it} + \gamma X_{it} + \delta M_{t} + \emptyset {\text{GFC}} + \varepsilon_{it }$$

where GFC refers to the global financial crisis. The variable GFC is a dummy variable that is equal to one for the GFC period (2007–2009)^6^ and zero if otherwise. The GFC period is consistent with prior banking literature [13, 41].

To empirically investigate the above models, we begin our analysis by applying the POLS method. The POLS technique enables us to obtain an overview of the nature of the relationship between dependent and independent variables. Further, this approach served as a baseline for the investigation of the models. Additionally, we assess both the contemporaneous and lagged effects of FinTech firms on bank stability. The use of the lagged values of the independent variables serves two purposes: First, it addresses the reverse causality concern, and second, it allows some time for the independent variables to impact the dependent variable. This is important for our study, as FinTech firms’ impact on bank stability may not be observed immediately. The use of lagged values of FinTech variables resolves this impact in relation to the time period.

Thereafter, we use the dynamic panel GMM estimator as a robustness check. This method is appropriate for our study context because, in the dynamic panel GMM model, first-differenced variables are used as instruments for the equations in levels, and the estimates are robust to unobserved heterogeneity, simultaneity, and dynamic endogeneity (if any). We follow this approach, as in Arellano and Bover [2] and Blundell and Bond [6]. This methodology is consistent with banking literature (e.g., [38, 40].

## Empirical results

### Preliminary analysis: descriptive statistics

We begin our preliminary investigation by presenting descriptive statistics for the variables in Table 1. The statistics suggest that the Z-score ranges from − 0.20 to 2.66 in the sample, which implies that financial stability varies significantly among the selected sample banks. Consistently, the relative financial stability measure (RZ-score) differs noticeably in the sample. Table 1 further suggests that FinTech measures exhibit higher standard deviations, as the number of these firms varies considerably across the sample period. The mean bank age was approximately 25 years, with the lowest being 10 years. The average NPL is nearly 5% of the total loans, with the highest at 73%. Another important indicator is asset growth, with an average of 11%. Notably, non-interest income (income diversity) is nearly 20% on average, which is a good sign indicating that banks are able to generate income from sources other than traditional banking services. However, it is equally important to note that the concentration of banks is extremely high in Malaysia, as only three banks represent over two-thirds of the entire banking system in the country. Overall, these descriptive statistics suggest significant variation in the sample observations.

**Table 1:** Descriptive statistics

Variable	Mean	Std. Dev	Min.	Max.
Z-score	1.61	0.50	− 0.20	2.66
RZ-score	0.80	0.13	0.31	0.96
FinTech	10.17	8.81	0.00	24.00
AFinTech	13.21	11.08	0.00	31.00
Bank size	3.80	0.78	0.47	5.29
Bank age	24.88	19.48	10.00	84.00
NPL ratio	4.67	7.10	0.00	73.31
Capitalisation	13.43	14.34	− 1.90	97.07
Assets growth	11.39	23.64	− 70.91	246.17
Income diversity	19.29	11.82	− 28.65	60.68
ROA	0.66	1.31	− 8.52	7.58
Bank concentration ratio	69.18	12.73	10.83	92.49
GDP growth rate	3.01	2.43	− 4.94	11.94
Bank-level corporate governance score	61.70	17.01	25.00	87.50

### Correlation matrix

A pairwise correlation matrix is presented in Appendix A1. The correlation matrix shows a positive correlation between financial stability (Z-score) and FinTech, indicating that the development of FinTech firms contributes to higher bank financial stability. The highest correlation between ROA and financial stability is 0.28. Therefore, multicollinearity is not a problem in this estimation. Finally, for the control variables, income diversity and bank-level governance exhibit a statistically significant positive correlation with financial stability, while bank concentration exhibits a significant negative correlation with financial stability. These significant associations between financial stability and the other control variables suggest the need to control for these variables in our regression model. Notably, none of the pairwise correlations among independent variables exceed 0.44 (between bank size and equity capital ratio), and all variance inflation factor values remain below 10 (untabulated). Thus, multicollinearity is unlikely to be a major concern in our study context.

### Main results and discussion

The empirical results are presented in Table 2. The first three columns explain the contemporaneous effect of FinTech firms on bank stability, while the last three columns provide information on the lagged effect (one year) of FinTech firms on the financial stability of banks. The results show that FinTech companies have significant positive impact on financial stability across alternative models. Notably, the lagged impact of FinTech companies on financial stability is greater, and this evidence is consistent across alternative models. Among the control variables, bank size, capitalization, and return on assets are statistically significant and play an important role in improving the financial stability of banks in Malaysia. Overall, these results suggest that the lagged impact of FinTech companies is greater on banks’ financial stability than the contemporaneous effect. These findings indicate that the banks begin responding and taking appropriate actions to improve their financial stability when they begin to experience the competition from the FinTech companies in the market.

**Table 2:** The effect of FinTech on financial stability

	Panel A: Contemporaneous effect of FinTech			Panel B: Lag effect of FinTech		
	DV: Financial stability					
	(1)	(2)	(3)	(4)	(5)	(6)
FinTech	0.0124*** (3.88)	0.0081** (2.41)	0.0067* (1.67)			
${\text{FinTech}}_{{{\text{t}} - 1}}$				0.0174*** (4.88)	0.0123*** (3.30)	0.0154*** (3.04)
Bank size		0.0680* (1.66)	0.0816** (1.98)		0.0844** (2.05)	0.0881** (2.13)
Bank age		− 0.0001 (− 0.07)	0.0003 (0.19)		0.0000 (0.01)	0.0002 (0.11)
NPL ratio		0.0004 (0.10)	− 0.0000 (− 0.01)		0.0000 (0.00)	− 0.0000 (− 0.00)
Capitalisation		0.0052** (2.33)	0.0053** (2.39)		0.0055** (2.29)	0.0051** (2.10)
Assets growth		− 0.0007 (− 0.56)	− 0.0006 (− 0.48)		− 0.0009 (− 0.57)	− 0.0010 (− 0.61)
Income diversity		0.0036 (1.49)	0.0041 (1.63)		0.0029 (1.12)	0.0035 (1.28)
ROA		0.1030*** (4.79)	0.1059*** (4.96)		0.1156*** (4.93)	0.1149*** (4.91)
Bank concentration ratio			− 0.0016 (− 0.56)			0.0028 (0.88)
GDP growth rate			0.0130 (1.09)			0.0065 (0.54)
Constant	1.4837*** (34.63)	1.0690*** (6.25)	1.0866***(4.04)	1.4509*** (33.72)	0.9798*** (5.58)	0.7116** (2.41)
*R*-squared	0.048	0.140	0.158	0.081	0.185	0.204
F-statistics	15.09***	5.86***	5.15***	23.85	7.39	6.34
Skewness and Kurtosis (Jarque–Bera) test for normality (*P*-value)	0.156	0.274	0.1093	0.148	0.210	0.1985
Observations	301	297	285	274	270	259

***, ** and * indicate statistical significance at 1%, 5% and 10% level respectively. T-stat are in parenthesis

In the next step of our investigation, we classified our sample banks into large and small groups. Table 3 reports the empirical results on the lagged effect of FinTech firms on financial stability by bank size. The results show that FinTech firms continue to positively impact the financial stability of large and small banks. However, FinTech firms’ influence is greater on the financial stability of small banks than on that of large banks. This empirical evidence makes more practical sense because small banks are more proactive due to their size and institutional setup to implement necessary actions to counter increasing competition and changing market conditions.

**Table 3:** The effect of FinTech on financial stability by bank size

	Panel A: Large banks			Panel B: Small banks		
	DV: Financial stability			DV: Financial stability		
	(1)	(2)	(3)	(4)	(5)	(6)
${\text{FinTech}}_{{{\text{t}} - 1}}$	0.0132*** (2.75)	0.0126** (2.31)	0.0161** (2.07)	0.0190*** (3.55)	0.0141** (2.60)	0.0174** (2.47)
Bank size		− 0.1021 (− 0.73)	− 0.1687 (− 1.16)		0.1221* (1.81)	0.1365** (2.00)
Bank age		− 0.0003(− 0.17)	0.0007(0.37)		0.0008 (0.29)	− 0.0002 (− 0.08)
NPL ratio		0.0006 (0.08)	− 0.0005 (− 0.07)		− 0.0014 (− 0.25)	− 0.0004 (− 0.07)
Capitalisation		0.0015 (0.26)	0.0033 (0.56)		0.0075** (2.49)	0.0069** (2.25)
Assets growth		0.0031 (0.94)	0.0015 (0.44)		− 0.0019 (− 0.93)	− 0.0015 (− 0.71)
Income diversity		0.0025 (0.66)	0.0025 (0.62)		0.0035 (0.93)	0.0041 (1.02)
ROA		0.1449*** (2.70)	0.1602*** (2.99)		0.1053*** (3.81)	0.1045*** (3.70)
Bank concentration ratio			0.0039 (0.72)			0.0023 (0.54)
GDP growth rate			− 0.0019 (− 0.09)			0.0078 (0.50)
Constant	1.5559*** (24.58)	1.7849*** (2.98)	1.7435** (2.37)	1.3728*** (23.56)	0.7997*** (3.08)	0.5476 (1.32)
*R*-squared	0.052	0.124	0.147	0.088	0.229	0.237
F-statistics	7.55***	2.26**	2.03**	12.59***	4.62***	3.69***
Observations	141	137	129	133	133	130

***, ** and * indicate statistical significance at 1%, 5% and 10% level respectively. T-stat are in parenthesis

We further conduct a sub-sample analysis based on bank-level corporate governance. Specifically, the sample banks in Malaysia are divided into high and low corporate governance groups, and the results are displayed in Table 4. Interestingly, the results show that FinTech companies have a greater positive impact on the financial stability of banks with low corporate governance than those with high corporate governance. The effects of the control variables were consistent, as shown in Table 3. The main takeaway from this analysis is that banks with low corporate governance are more proactive in improving their financial stability with the increasing presence of FinTech companies. This suggests that low corporate governance banks adopt more FinTech in their banking services, which could be characterized as a behavior to mask poor governance or take advantage of the weak governance framework of FinTech practices in banking sector.

**Table 4:** The effect of FinTech on financial stability by bank-level corporate governance

	Panel A: High corporate governance			Panel B: Low corporate governance		
	DV: Financial stability			DV: Financial stability		
	(1)	(2)	(3)	(4)	(5)	(6)
${\text{FinTech}}_{{{\text{t}} - 1}}$	0.0106* (1.66)	0.0132* (1.75)	0.0118 (0.99)	0.0199*** (4.92)	0.0135*** (3.41)	0.0162*** (3.16)
Bank size		− 0.0311 (− 0.26)	− 0.0576 (− 0.43)		0.1024** (2.46)	0.1058** (2.48)
Bank age		− 0.0021 (− 0.83)	− 0.0017 (− 0.63)		0.0012 (0.63)	0.0011 (0.53)
NPL ratio		− 0.0021 (− 0.22)	− 0.0028 (− 0.30)		0.0001 (0.02)	0.0008 (0.20)
Capitalisation		0.0046 (0.63)	0.0036 (0.42)		0.0049** (2.23)	0.0049** (2.19)
Assets growth		0.0036 (0.83)	0.0037 (0.84)		− 0.0015 (− 0.94)	− 0.0016 (− 0.97)
Income diversity		− 0.0026 (− 0.50)	− 0.0021 (− 0.37)		0.0055** (2.02)	0.0063** (2.16)
ROA		0.1294 (0.93)	0.1812 (1.24)		0.1080*** (5.25)	0.1074*** (5.13)
Bank concentration ratio			− 0.0017 (− 0.21)			0.0024 (0.73)
GDP growth rate			0.0092 (0.37)			0.0118 (0.88)
Constant	1.6226*** (19.66)	1.6482*** (3.30)	1.7897* (1.91)	1.3608*** (29.22)	0.8176*** (4.80)	0.5694** (2.02)
*R*-squared	0.025	0.058	0.067	0.130	0.318	0.329
F − statistics	2.76*	1.76*	1.65*	24.20***	8.87***	7.20***
Observations	110	109	101	164	161	158

***, ** and * indicate statistical significance at 1%, 5% and 10% level respectively. T-stat are in parenthesis

We further perform a sub-sample analysis by bank type. Table 5 presents the results of FinTech companies’ impact on the financial stability of Islamic and conventional banks. The findings show that FinTech companies have a greater positive and significant impact on the financial stability of Islamic banks than on that of conventional banks. This indicates that Islamic banks are more efficient in adopting FinTech technologies in their services and in managing the competition arising from FinTech firms. We further investigate FinTech companies’ impact on banks’ financial stability by controlling for the GFC period, the results of which are displayed in Table 6. The findings reveal that FinTech companies continue to positively drive the financial stability of banks in Malaysia. We further note that bank size, capitalization, and return on assets are the other potential drivers of banks’ financial stability.

**Table 5:** The effect of FinTech on financial stability by bank type (Islamic vs. conventional)

	Panel A: Islamic banks			Panel A: Conventional banks		
	DV: Financial stability			DV: Financial stability		
	(1)	(2)	(3)	(4)	(5)	(6)
${\mathrm{FinTech}}_{\mathrm{t}-1}$	0.0260*** (6.17)	0.0208*** (4.87)	0.0226*** (4.25)	0.0082 (1.56)	0.0125* (1.90)	0.0100 (0.97)
Bank size		0.0631 (1.53)	0.0637 (1.53)		− 0.0508 (− 0.43)	− 0.0845 (− 0.64)
Bank age		− 0.0021 (− 0.75)	− 0.0020 (− 0.71)		− 0.0026 (− 1.24)	− 0.0023 (− 1.05)
NPL ratio		− 0.0005 (− 0.10)	0.0000 (0.01)		0.0006 (0.09)	− 0.0002 (− 0.03)
Capitalisation		0.0051** (2.18)	0.0052** (2.17)		− 0.0004 (− 0.07)	− 0.0023 (− 0.33)
Assets growth		− 0.0029* (− 1.73)	− 0.0030* (− 1.75)		0.0061* (1.84)	0.0058* (1.66)
Income diversity		0.0016 (0.57)	0.0019 (0.65)		− 0.0012 (− 0.25)	− 0.0025 (− 0.43)
ROA		0.0906*** (4.46)	0.0903*** (4.41)		0.0802 (0.62)	0.1142 (0.82)
Bank concentration ratio			0.0016 (0.51)			− 0.0028 (− 0.39)
GDP growth rate			0.0036 (0.30)			0.0192 (0.73)
Constant	1.2296*** (24.50)	0.9948*** (5.76)	0.8479*** (2.85)	1.6846*** (26.26)	1.8524*** (3.83)	2.1648** (2.52)
*R*-squared	0.218	0.361	0.363	0.018	0.059	0.071
F-statistics	38.09***	9.19***	7.31***	2.44*	0.95	0.83
Observations	139	139	139	135	131	120

***, ** and * indicate statistical significance at 1%, 5% and 10% level respectively. T-stat are in parenthesis

**Table 6:** The effect of FinTech on financial stability controlling for GFC

	DV: Financial stability		
	(1)	(2)	(3)
${\mathrm{FinTech}}_{\mathrm{t}-1}$	0.0157*** (4.14)	0.0112*** (2.86)	0.0146*** (2.67)
Bank size		0.0835** (2.03)	0.0877** (2.12)
Bank age		0.0000 (0.00)	0.0002 (0.12)
NPL ratio		0.0004 (0.11)	0.0001 (0.03)
Capitalisation		0.0053** (2.19)	0.0050** (2.04)
Assets growth		− 0.0009 (− 0.56)	− 0.0010 (− 0.60)
Income diversity		0.0029 (1.13)	0.0034 (1.27)
ROA		0.1142*** (4.87)	0.1145*** (4.88)
Bank concentration ratio			0.0026 (0.79)
GDP growth rate			0.0043 (0.32)
GFC	− 0.1062 (− 1.21)	− 0.0810 (− 0.95)	− 0.0386 (− 0.39)
Constant	1.4810*** (29.83)	1.0062*** (5.66)	0.7502** (2.40)
*R*-squared	0.086	0.187	0.204
F-statistics	12.68***	6.66***	5.76***
Observations	274	270	259

***, ** and * indicate statistical significance at 1%, 5% and 10% level respectively. T-stat are in parenthesis

## Robustness tests

We conduct further investigation using alternative measures of financial stability (a relative measure using the stochastic frontier approach) and FinTech (firms that offer banking services as well as wealthtech, remittance/FX, regtech, insurtech, and proptech). The use of a stochastic frontier-based financial stability measure accommodates the relative financial stability measure and ranks a bank’s financial stability relative to the optimal financially stable banks in the entire industry. Table 7 presents the results of the alternative measures of financial stability. The results suggest that FinTech companies continue to significantly positively impact banks’ financial stability. The effect size is economically significant and robust across the models. This evidence demonstrates that irrespective of the measures of financial stability, FinTech firms positively impact bank financial stability.

**Table 7:** The effect of FinTech on financial stability: Alternative measure of financial stability

	DV: Relative financial stability (RZ-score)		
	(1)	(2)	(3)
${\mathrm{FinTech}}_{\mathrm{t}-1}$	0.0016* (1.83)	0.0030* (1.85)	0.0015*** (2.66)
GFC	0.0578** (2.02)	0.0237 (0.53)	0.0230 (0.40)
Bank size		− 0.0466 (− 1.33)	− 0.0450 (− 1.10)
Bank age		− 0.0002 (− 0.33)	− 0.0000 (− 0.01)
NPL ratio		− 0.0001 (− 0.05)	− 0.0004 (− 0.22)
Capitalisation		0.0000 (0.02)	0.0001 (0.04)
Assets growth		0.0010 (0.89)	0.0009 (0.80)
Income diversity		− 0.0004 (− 0.28)	− 0.0003 (− 0.17)
ROA		0.0386 (0.91)	0.0520 (1.16)
Bank concentration ratio			− 0.0016 (− 0.85)
GDP growth rate			0.0044 (0.39)
Constant	0.7775*** (59.73)	0.9532*** (6.53)	1.0374*** (4.10)
*R*-squared	0.024	0.055	0.055
F-statistics	3.01**	2.70**	9.50***
Observations	274	270	259

***, ** and * indicate statistical significance at 1%, 5% and 10% level respectively. T-stat are in parenthesis

We further examine FinTech firms’ impact on bank financial stability using an alternative measure of FinTech. In the previous sections, we used FinTech firms closely related to the banking industry as our primary FinTech measure. In this section, we expand our measure by accounting for all FinTech firms to understand their overall impact, the results of which are reported in Table 8: The table shows that the alternative FinTech indicator is positive and statistically significantly impacts the financial stability of banks in Malaysia. Notably, the effect size is less pronounced with this alternative measure of FinTech compared with our main results, as reported in Table 3. This evidence further suggests that FinTech firms that are closely related to the banking industry have a greater influence on bank financial stability than other FinTech firms that are not closely related to the banking industry.

**Table 8:** The effect of FinTech on financial stability: Alternative proxy for FinTech

	DV: Financial stability		
	(1)	(2)	(3)
${\mathrm{AFinTech}}_{\mathrm{t}-1}$	0.0128*** (4.66)	0.0087*** (3.04)	0.0110***(2.79)
Bank size		0.0852** (2.06)	0.0890** (2.15)
Bank age		0.0005 (0.30)	0.0008 (0.47)
NPL ratio		− 0.0002 (− 0.04)	− 0.0002 (− 0.04)
Capitalisation		0.0057** (2.35)	0.0053** (2.17)
Assets growth		− 0.0007 (− 0.43)	− 0.0007 (− 0.42)
Income diversity		0.0029 (1.14)	0.0035 (1.29)
ROA		0.1162*** (4.94)	0.1157***(4.92)
Bank concentration ratio			0.0024 (0.74)
GDP growth rate			0.0081 (0.68)
Constant	1.4545*** (33.31)	0.9664*** (5.49)	0.7203** (2.38)
*R*-squared	0.074	0.180	0.199
F-statistics	21.72***	7.15***	6.16***
Observations	274	270	259

***, ** and * indicate statistical significance at 1%, 5% and 10% level respectively. T-stat are in parenthesis

Finally, using alternative estimation techniques such as the dynamic panel GMM, we estimate the models and report the results in Table 9. The results show that FinTech companies continue to positively influence financial stability, and the result is consistent with the alternative measure of financial stability. The robust result obtained using the dynamic panel GMM estimator confirms that our result is not driven by unobserved heterogeneity, simultaneity, and dynamic endogeneity. Taken together, our robust evidence shows that FinTech firms and bank financial stability are positively and significantly associated.

**Table 9:** The effect of FinTech on financial stability: Dynamic panel GMM estimation results

	DV: Financial stability	DV: Relative Financial stability
	(1)	(2)
${\mathrm{Zscore}}_{\mathrm{t}-1}$	0.3392 (1.59)	
${\mathrm{Refs}}_{\mathrm{t}-1}$		0.5170 (0.69)
${\mathrm{FinTech}}_{\mathrm{t}-1}$	0.0113** (2.54)	0.0105** (2.09)
Bank size	0.2976** (2.34)	0.4756** (2.33)
Bank age	0.0040 (0.30)	− 0.0034 (− 0.38)
NPL ratio	− 0.0056 (− 0.27)	− 0.0143 (− 0.82)
Capitalisation	0.0068** (2.58)	0.0108** (1.82)
Assets growth	− 0.0035 (− 0.44)	− 0.0101 (− 0.84)
Income diversity	− 0.0095 (− 0.47)	− 0.0028 (− 0.28)
ROA	− 0.0376 (− 0.33)	0.0221 (0.18)
Bank concentration ratio	0.0024 (0.46)	0.0019 (0.38)
GDP growth rate	− 0.0044 (− 0.27)	0.0087 (0.61)
Constant	− 0.2680 (− 0.39)	− 0.7267 (− 0.96)
F-statistics	8.14***	7.27***
Hansen J statistics (*P*-value)	0.774	0.972
AR (1) test	− 2.01**	− 1.96**
AR (2) test	1.06	0.16
Observations	259	259

***, ** and * indicate statistical significance at 1%, 5% and 10% level respectively. T-stat are in parenthesis

To account for the rapid changes in FinTech development in the post-2010 period, we examine FinTech firms’ impact on bank financial stability in this period. The empirical results are presented in Table 10. We find evidence that FinTech firms positively impact bank financial stability. This result is robust across models, suggesting that FinTech contributes to improving banks’ financial stability.

**Table 10:** The effect of FinTech on financial stability: Post 2010

	DV: Financial stability		
	(1)	(2)	(3)
${\mathrm{FinTech}}_{\mathrm{t}-1}$	0.0137*** (2.74)	0.0116** (2.24)	0.0088 (1.30)
Bank size		0.0827* (1.76)	0.0894* (1.88)
Bank age		− 0.0003 (− 0.15)	− 0.0002 (− 0.08)
NPL ratio		0.0017 (0.24)	0.0016 (0.22)
Equity capital ratio		0.0042 (1.52)	0.0041 (1.45)
Assets growth		− 0.0017 (− 0.86)	− 0.0011 (− 0.53)
Income diversity		0.0009 (0.28)	0.0006 (0.18)
ROA		0.0748** (2.06)	0.0807** (2.22)
Bank concentration ratio			− 0.0038 (− 0.96)
GDP growth rate			0.0168 (0.87)
Constant	1.5006*** (22.37)	1.0871*** (5.03)	1.2810*** (3.36)
*R*-squared	0.037	0.086	0.104
F-statistics	7.48	2.17	2.00
Observations	198	194	183

## Conclusion

Overall, our empirical results show that Fintech companies have a significant positive impact on the financial stability of banks in Malaysia. Our study further demonstrates that Fintech firms exhibit a more positive influence on the financial stability of small banks, low corporate governance banks, and Islamic banks. Given these findings, we offer several policy implications that are crucial for further strengthening the financial stability of banks in Malaysia. Specifically, we argue that large banks are not as quick as small banks in implementing appropriate actions to improve their financial stability. Hence, we suggest that large banks continue to protect their customer base by offering competitive and innovative services to meet the expectations of customers in the market. This recommendation is consistent with the findings of Chen et al. [8], who argue that financial institutions can avoid the negative impact of competitor innovations by investing heavily in their own innovations. By doing so, banks can compete with their counterparts to offer more effective and competitive financial services to customers. The same argument also applies to high corporate governance banks and conventional banks, which are slightly lacking in improving their financial stability as compared with their counterparts with respect to the increasing presence of FinTech companies in the country.

Further, we suggest that Malaysian banks’ financial stability, in general, has increased with the presence of FinTech companies; however, it varies considerably across banks. FinTech firms continue to grow due to the changing circumstances in the economy (e.g., COVID-19), technological innovations, and convenient and low-cost financial services. Thus, banks must be ready to protect their customer base and market power by providing competitive and attractive services to the customers. This study’s main takeaway is that FinTech companies’ presence does not negatively influence banks’ financial stability, creates healthy market competition, and may improve banking services for unbanked customers. Finally, we suggest that future studies may try investigating COVID-19’s impact on banks’ financial stability as well as FinTech firms’ growth. Future studies may also examine FinTech firms’ impact on both banking and non-banking firms, thereby further enhancing our understanding of FinTech and its wider impact.

## Appendix

See Table

**Table 11:** Correlation matrix

	(1)	(2)	(3)	(4)	(5)	(6)	(7)	(8)	(9)	(10)	(11)	(12)
Z-score (1)	1.0											
FinTech (2)	0.21*	1.0										
Bank size (3)	0.10	0.08	1.0									
Bank age (4)	0.05	0.08	0.27*	1.0								
NPL ratio (5)	− 0.02	− 0.16*	− 0.06	0.06								
Capitalisation (6)	0.06	0.13*	− 0.44*	− 0.02	1.0	1.0						
Assets growth (7)	− 0.09	− 0.18*	− 0.08	− 0.04	0.05	0.06	1.0					
Income diversity (8)	0.13*	0.17*	0.21*	0.19*	0.06	− 0.11	− 0.11	1.0				
ROA (9)	0.28*	0.11	0.11	0.08	− 0.04	− 0.16*	− 0.13*	0.03	1.0			
Bank concentration ratio (10)	− 0.13*	− 0.60*	− 0.04	− 0.09	0.07	− 0.04	0.14*	− 0.17*	− 0.01	1.0		
GDP growth rate (11)	0.06	0.03	− 0.01	0.03	− 0.07	− 0.03	0.01	− 0.04	0.04	0.26*	1.0	
Bank-level governance score (12)	0.23*	0.06	0.28*	0.21*	− 0.02	− 0.10	− 0.15*	0.19*	0.18*	0.02	0.11	1.0

^*^Indicates statistical significance at 5% level

11.

## Footnotes

1 See Fin-Tech Global and Accenture global boom in FinTech investment.

2 Notably, the Chinese financial system does not have a fully developed system of credit referencing.

3 In recent time, there is an increasing literature on bitcoin/cryptocurrency and linking it to various financial measures (e.g. [21, 25, 26, 33–35].

4 See The Global FinTech Index 2020 as published by Findexable ltd., https://findexable.com/wp-content/uploads/2019/12/Findexable_Global-Fintech-Rankings-2020exSFA.pdf

5 FinTech Malaysia presents FinTech firms’ details, which can be accessed at: https://fintechnews.my/list-fintech-startup-malaysia-fintech-companies-malaysia-directory/

6 The selection of the GFC period is based on previous literature (e.g., [37].
